# Turban PUVASOL: An Effective Treatment in Alopecia Totalis

**DOI:** 10.4103/0974-7753.77520

**Published:** 2010

**Authors:** L Sornakumar, C Shanmuga Sekar, CR Srinivas

**Affiliations:** Department of Dermatology, PSG Hospitals, Coimbatore, Tamil Nadu, India

**Keywords:** Alopecia totalis, PUVASOL, Turban

## Abstract

Alopecia areata is characterized by patchy hair loss involving the scalp, eyelashes, and beard. The disease may at times lead to complete baldness of the scalp (alopecia totalis) or of the entire body (alopecia universalis). Alopecia totalis is usually resistant to therapy. We report two cases of alopecia totalis treated with turban psoralen with sunlight exposure (PUVASOL).

## INTRODUCTION

Alopecia totalis is a distressing problem which is usually resistant to various therapeutic modalities. PUVA therapy is an effective therapeutic option but is associated with side effects such as nausea, requires eye protection, and cannot be used in children. Through use of ‘turban PUVASOL’, it is now possible to administer a dilute bathwater solution containing 8-methoxypsoralen (8-MOP) to the scalp. We report two cases of alopecia totalis which responded to turban PUVASOL (psoralen with sunlight exposure).

## CASE REPORTS

### Case 1

**Figure 1:**
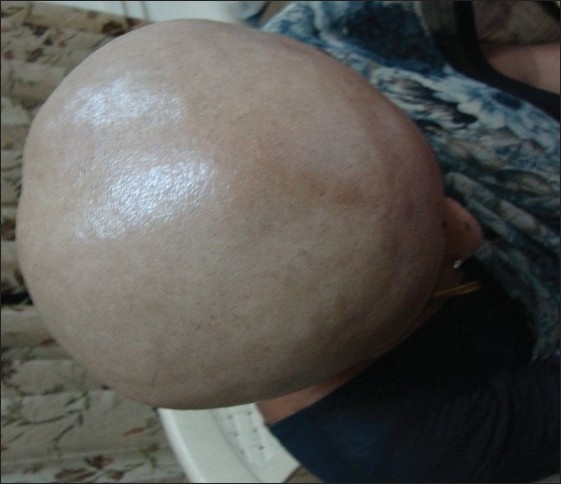
Complete loss of scalp hair

**Figure 2:**
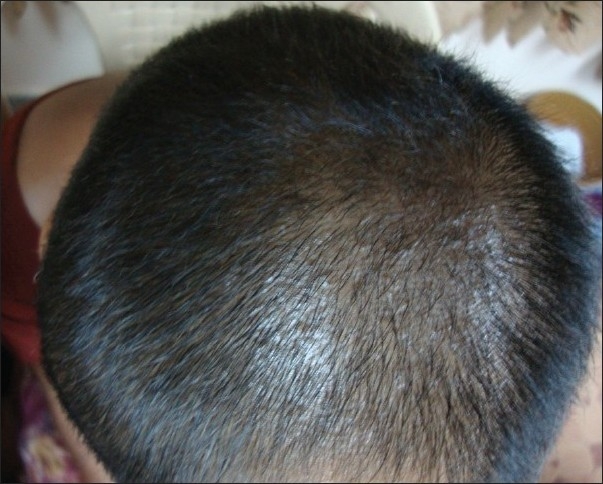
Regrowth of scalp hair after 6 months

A 41-year-old woman presented with complete loss of scalp hair since 12 months [Figure 1]. The diagnosis was confirmed by biopsy. She was treated with minoxidil 10% twice daily and 4 mg of betamethasone twice weekly for 6 months with no significant response. She was further treated with turban PUVASOL therapy as described below. After 2 months, vellus hair started growing and by 4 months, she had significant hair growth [Figure 2].

In the turban PUVASOL therapy, 1 ml of 8-methoxypsoralen (8-MOP) 1% was diluted in 2 l of water. A clean absorbent cotton cloth was soaked into the solution for 30 seconds and wrapped around the scalp in a turban-like fashion for 5 minutes. The process was repeated four times. The patients were further exposed to sunlight for 5 minutes and the time was gradually increased up to 15 minutes. The treatment was carried out thrice a week. The time of exposure was between 10:00 and 10:15 AM when the ultraviolet (UV)A : UVB ratio is maximum. The patient was advised to avoid sun exposure for few hours after treatment.

### Case 2

An 8-year-old boy presented with complete loss of hair over the scalp since 20 months. The diagnosis was confirmed by histopathology. He was treated for 1 year with 2 mg of betamethasone twice weekly and minoxidil 5% twice daily with no significant response. We decided to treat the patient with turban PUVASOL therapy similar to previous case. The patient started having vellus hair growth after 2 months and by 6 months there was almost complete regrowth of scalp.

## DISCUSSION

Alopecia areata (AA) is an autoimmune disease characterized by patchy, nonscarring hair loss. Although it most commonly affects the scalp, it can involve any hair-bearing area on the body. Alopecia totalis refers to complete loss of scalp hair. AA can lead to severe emotional distress. Various therapeutic modalities such as systemic steroids, contact immunotherapy, and phototherapy have been tried with partial success.

In 1974, Rollier and Warcewski[1] induced hair regrowth in a patient with AA using 8-MOP and natural sunlight. The first report of PUVA treatment with artificial UVA light for AA was published in 1978 by Weissman *et al*.[2] who obtained encouraging results in five patients.

The turban PUVA can be considered a variation of the so called bath suit PUVA method as described by Pai and Srinivas.[3] It has the advantage of delivering the desired photosensitizing effect in a localized area without systemic side effects. Behrens-Williams *et al*.[4] reported hair regrowth in six of nine patients after 10 weeks of treatment. Broniarczyk-Dyla *et al*.[5] observed that turban PUVA was significantly more effective in AA vulgaris than in AA totalis or universalis and proved to be a safe, well-tolerated method without the systemic side effects of PUVA.

Our patients were not able to come regularly for turban PUVA treatment and hence we decided to modify it as turban PUVASOL. The mechanism of action of PUVA in AA is not clearly understood. There is circumstantial and indirect evidence that AA is a T cell-mediated autoimmune disease of the hair follicle. Immunohistochemical studies in human beings and animal models have demonstrated a perifollicular accumulation of dendritic cells plus CD4+ and CD8+ T lymphocytes and isolated polymorphonuclear cells.[6] Because dendritic cells are thought to play an important role in the initiation of AA by antigen presentation and costimulation, PUVA treatment might exert an inhibitory effect on the development of AA. Suppression of IL-2 and IL-1ß production might suppress an already present lymphocytic infiltrate in AA and induce hair growth.

The time chosen for phototherapy was between 10:00 and 10:15 AM. Balasaraswathy *et al*.[7] in a study proved that the ideal time for PUVASOL is between 9:15 and 11:15 AM, which will minimize unwanted exposure to UVB and infrared radiation and hence we regularly advise this particular time in our institute. Both our patients had significant improvement after therapy. We report this case as the effectiveness of turban PUVASOL in AA has never been reported.
